# Photoswitchable Fluorescent Proteins: Mechanisms on Ultrafast Timescales

**DOI:** 10.3390/ijms23126459

**Published:** 2022-06-09

**Authors:** Longteng Tang, Chong Fang

**Affiliations:** Department of Chemistry, Oregon State University, 153 Gilbert Hall, Corvallis, OR 97331-4003, USA

**Keywords:** photoswitchable fluorescent proteins, ultrafast techniques, *cis*-*trans* isomerization, proton transfer, molecular reaction dynamics, excited state processes, photochemistry

## Abstract

The advancement of super-resolution imaging (SRI) relies on fluorescent proteins with novel photochromic properties. Using light, the reversibly switchable fluorescent proteins (RSFPs) can be converted between bright and dark states for many photocycles and their emergence has inspired the invention of advanced SRI techniques. The general photoswitching mechanism involves the chromophore *cis*-*trans* isomerization and proton transfer for negative and positive RSFPs and hydration–dehydration for decoupled RSFPs. However, a detailed understanding of these processes on ultrafast timescales (femtosecond to millisecond) is lacking, which fundamentally hinders the further development of RSFPs. In this review, we summarize the current progress of utilizing various ultrafast electronic and vibrational spectroscopies, and time-resolved crystallography in investigating the on/off photoswitching pathways of RSFPs. We show that significant insights have been gained for some well-studied proteins, but the real-time “action” details regarding the bidirectional *cis*-*trans* isomerization, proton transfer, and intermediate states remain unclear for most systems, and many other relevant proteins have not been studied yet. We expect this review to lay the foundation and inspire more ultrafast studies on existing and future engineered RSFPs. The gained mechanistic insights will accelerate the rational development of RSFPs with enhanced two-way switching rate and efficiency, better photostability, higher brightness, and redder emission colors.

## 1. Introduction

Fluorescent protein (FP)-based microscopy and nanoscopy have revolutionized the bioimaging and life sciences for the past three decades [1,2,3,4]. On the imaging front, the commonly encountered low spatial resolution (~200 nm) of conventional fluorescence microscopy limits its applications in the detection of subcellular structures. Because of the development of various FP-based optical highlighters, super-resolution imaging has emerged to overcome the diffraction barrier, culminating in the 2014 Nobel Prize in Chemistry [5,6,7,8,9,10]. Among three types of optical highlighters, the photoswitchable fluorescent proteins, also known as reversibly switchable fluorescent proteins (RSFPs), have the advantage of switching back and forth between bright and dark states for multiple photocycles [11,12]. In contrast, the fluorescence of photoconvertible and photoactivatable FPs can only be transformed to a different color or bright state irreversibly [13,14].

Since the discovery of the first RSFP called asFP595 (tetrameric) in 2000 [15], many RSFPs with monomeric states, long emission wavelengths, high photostability, fast maturation, and high photoswitching quantum yield (PQY) have been developed [16,17,18,19]. In general, they can be classified into three categories: negative, positive, and decoupled types [20]. For most RSFPs, the photoswitching light and the fluorescence-excitation light are coupled. For a negative RSFP such as Dronpa [21], the light used to induce fluorescence can often switch the protein from the on to off state; whereas for a positive RSFP like Padron [22], the same light can turn more proteins on. In contrast, the decoupled RSFPs can use separate light with different wavelengths for fluorescence excitation and photoswitching [11]. Notably, almost all known RSFPs are either negative or positive types while the decoupled RSFPs have been rarely discovered. To date, Dreiklang and SPOON represent the only two reported decoupled RSFPs [23,24], and none has been reported to be engineered from the positive or negative RSFPs, substantiating their system-dependent nature (e.g., no cross over between the negative/positive and decoupled RSFP categories) that warrants all the individual studies as reviewed in Section 2, Section 3 and Section 4 below.

**Figure 1:**
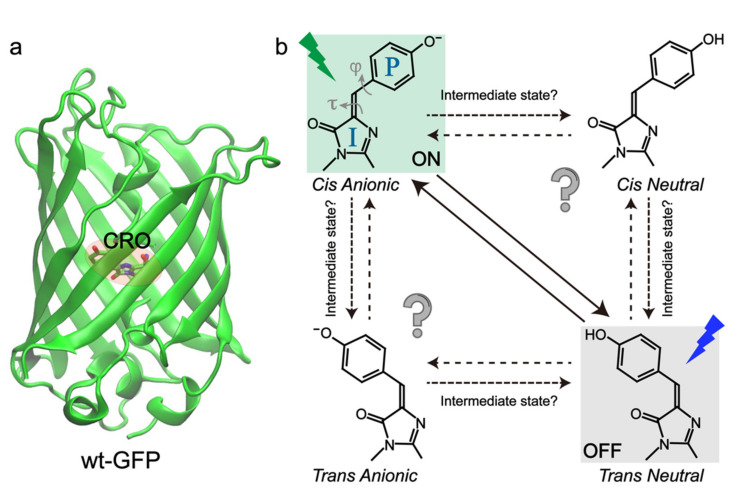
Schematic of the overall structure and photoswitching mechanisms of RSFPs. (**a**) Crystal structure of the wild-type green fluorescent protein (wtGFP) (PDB ID: 1EMA) [25]. (**b**) The photoinduced structural and protonation state changes of the chromophore (P- and I-rings denoted in the “ON” state) during reversible switching. Possible transitions and associated intermediate states are indicated by a network of solid and dashed arrows.

RSFPs typically adopt a soda-can structure similar to wild-type green fluorescent protein (wtGFP) with the chromophore (CRO) embedded at the β-barrel center (Figure 1a), which can be modeled as *p*-hydroxybenzylidene-2,3-dimethylimidazolinone (*p*-HBDI) [25,26,27,28]. To uncover the structure of RSFPs in bright and dark states, X-ray crystallography, steady-state electronic spectroscopy, and molecular dynamics (MD) simulations have been widely implemented [29,30,31,32,33,34,35]. The current consensus about photoswitching pathways is that they involve the *cis*-*trans* isomerization and protonation state change of the chromophore for most RSFPs (Figure 1b), except the decoupled ones. We note that the *cis*-*trans* isomerization has been extensively studied in the gas phase for molecules like azobenzene and retinal, especially on the femtosecond (fs) to picosecond (ps) timescales, but the retrieved mechanistic information cannot be simply transferred to the condensed phases (e.g., a protein matrix) due to the local environment change and the resultant difference in molecular Hamiltonian and density matrix. During the chromophore transformation, the surrounding protein residues also need to relocate or reorient to accommodate the chromophore [11,20]. However, only knowing the protein structures at the starting and ending states of the photoswitching events is not sufficient. Key information regarding the detailed switching routes after photoexcitation is still lacking, which lies at the molecular level to fundamentally determine the switching rate and efficiency.

In particular, the intrinsic (electronic and atomic) motions of the “light-harvesting” chromophore are of great interest because, as the photosensory unit, it functions as a molecular-level “machine” that can absorb photon energy and convert it to mechanical motions. Some of the pertinent questions require further investigations (Figure 1b) that will benefit from continuing experimental, theoretical, and computational advances. For example, although *cis*-*trans* isomerization and proton transfer are involved during the photoswitching events, their sequence, associated electronic states and timescales have been constantly under debate. The exact twisting coordinates (dihedral angles, one-bond flip, or hula twist) and geometries and whether intermediate states are involved remain elusive for most RSFPs [36,37,38]. Studies on photoisomerization events have been a hot topic for the HBDI chromophore and its derivatives in solution, but the gained knowledge cannot be directly transferred into a chromophore in the protein matrix due to the complex electrostatic and steric interactions between the chromophore and surrounding protein residues, which are intrinsically more heterogeneous and asymmetric than the solution environment [39,40,41,42]. Therefore, it is urgent and necessary to elucidate the detailed structural motions of the chromophore and local amino acids in the electronically excited and “hot” (non-equilibrium) ground states, which are essential for the deepened understanding and rational development of next-generation RSFPs [43].

During the past decade, researchers have started to implement more ultrafast techniques that include time-resolved electronic and vibrational spectroscopies as well as femtosecond crystallography to map the detailed photoswitching pathways [44,45,46]. The retrieved mechanistic information has benefited the efficient development of new RSFPs, such as an rsEGFP2 mutant [45]. To date, there has not been a systematic review of these evolving results. In this contribution, we summarize the recent progress of implementing various ultrafast techniques in unveiling the switching mechanism of RSFPs from femtosecond (fs) to millisecond (ms) timescales based on the protein type, with relevant discussions and concluding remarks in the end. By offering this timely review, we aim to lay a solid foundation and provide useful guidance for future ultrafast studies on RSFPs.

## 2. Negative RSFPs

### 2.1. Dronpa and Dronpa-2

Dronpa is the first monomeric negative RSFP engineered from coral *Pectiniidae* by Miyawaki et al. in 2004 [21]. It adopts an autocatalytically formed Cys62-Tyr63-Gly64 (CYG) chromophore [32]. In the resting state at pH 7.4, the majority of Dronpa is in a bright/on state with a dominant absorption peak at 503 nm, corresponding to the *cis*-anionic chromophore [47,48,49]. A minor absorption peak at 390 nm is due to the neutral chromophore. Irradiation at 490 nm generates strong fluorescence centered at 518 nm with a high fluorescence quantum yield (FQY) of 0.85. The 490 nm light can also transform Dronpa from the native on state to a dark/off state, adopting the *trans*-neutral chromophore, with a measured photoswitching quantum yield (PQY) of 3.2 × 10^−4^ [32,50]. A 400 nm light quickly converts the protein to its on state with an off-to-on PQY of 0.37 [21]. The development of Dronpa made it possible to track fast protein dynamics in living cells. To improve the PQY of Dronpa, rsFastLime was developed via a single V157G mutation to reduce steric opposition to a *trans* chromophore [48,51]. It displays a much faster on-to-off switching rate and PQY (3.5 × 10^−3^) and only a small sacrifice of the FQY (0.77) versus Dronpa, making it a promising tool for bioimaging applications [48,52]. Dronpa-2 and Dronpa-3 were then developed with the single (M159T) and double (V157I and M159A) mutations with even higher on-to-off PQY values of 4.7 × 10^−2^ and 5.3 × 10^−3^, respectively [53]. They were also able to quickly return to their bright state under dark adaptation. Notably, the on-to-off and off-to-on switching speeds of Dronpa-2 are more than 1100- and 2-fold faster than the rates of Dronpa in whole-cell imaging measurements [48]. However, these mutations come with a significant sacrifice of the FQYs (0.33 [53] or 0.23 [48] for Dronpa-2 and 0.28 [53] for Dronpa-3), which is understandable due to the ultrafast competition nature between the chromophore radiative and nonradiative pathways after photoexcitation [39]. The low FQY/brightness of Dronpa-2 limits its application in cellular imaging but its high PQY and switching rates render it suitable for ultrafast studies.

The first picosecond (ps) investigation of Dronpa in buffer solutions was performed by Habuchi et al. in 2005, using time-resolved fluorescence with single photon counting technique [54]. They discovered that Dronpa in the on state has a long lifetime of 3.6 nanoseconds (ns) following photoexcitation at 488 nm, in accord with its high FQY (0.85). The low-pH-induced protonated form can quickly return to the ground state with an average time constant of ~14 ps after 390 nm excitation in aqueous buffer at pH 7.4. Unlike the photoinduced protonated form, this low-pH-induced chromophore population lacks the photoconversion capability. On the single-molecule level, bright Dronpa can be switched to a dark state which has a 65 ms lifetime to return to the on state. This study pinpointed characteristic photoswitching timescales and laid the foundation for future ultrafast investigations. Given that the retrieved time constants span from ps to ms, ultrafast techniques with fs resolution and ms detection window are needed for an in-depth mechanistic investigation, especially implementing the experimental toolsets that are structurally sensitive (e.g., vibrational spectroscopies and crystallography).

Two years later, Fron et al. implemented fs transient absorption (fs-TA) with a 395 nm photoexcitation pulse to target the off-to-on photoswitching process of Dronpa [44]. By contrasting the fs-TA spectral dynamics between the low-pH-induced and photoinduced protonated forms around the 420 nm probe region (following 395 nm excitation in aqueous buffer at pH 8.0), they discovered that the photoconverted protonated form in the off state exhibits a unique 4 ps process, which was attributed to excited-state proton transfer (ESPT) as supported by the observed deuterium (D)/hydrogen (H) kinetic isotope effect of ~2. However, this particular assignment was based on the dynamics of a rather noisy TA band in a highly overlapped region between the excited-state absorption (ESA) band of the deprotonated chromophore around 420 nm and the stimulated emission (SE) band of the protonated chromophore species around 450 nm [54], so its probe-dependent fits were likely convoluted and complex to interpret. In addition, the 4 ps component was characterized as a decay process; if the 420 nm signal indeed originated from the ESA band of a deprotonated chromophore, a prominent ESPT step would lead to a rise component at this wavelength on the aforementioned timescale. The ultrafast ESPT argument was later challenged by several ultrafast studies (see below). Moreover, this study [44] offered little information about the protein structural evolution.

**Figure 2:**
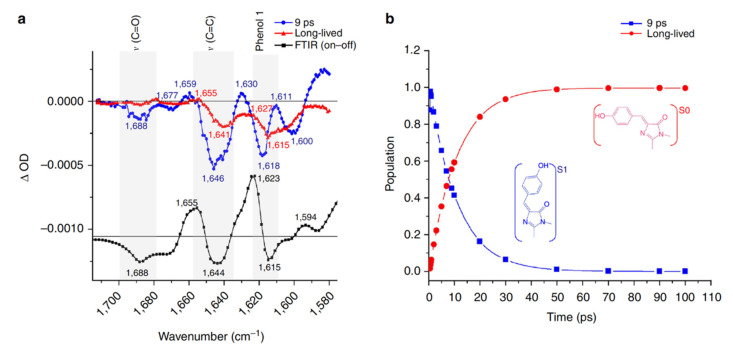
Time-resolved infrared (IR) spectroscopy reveals characteristic species during the off-to-on photoswitching of Dronpa after 400 nm excitation. (**a**) Time-resolved difference spectra (pump-on minus pump-off) retrieved from global analysis with a sequential model (blue→red) and the FTIR difference spectra between the on and off states in D_2_O (black). (**b**) Global fitting results of the short-lived (blue) and long-lived (red) species with a dominant 9 ps component due to the decay of the singlet excited state of the *trans*-neutral chromophore (blue structure shown in the inset). Reprinted with permission from Ref. [55]. Copyright 2013 The Authors.

In 2013, the off-to-on photoactivation of Dronpa was revisited by Warren et al. with a fs vibrational technique, time-resolved infrared (TRIR) spectroscopy, for the first time [55]. They focused on the high-frequency vibrational region of the photoswitched Dronpa after 400 nm excitation in aqueous buffer solution at pH or pD 7.8. Global analysis of the TRIR spectra with a sequential model revealed a short-lived species with a 9 ps lifetime and a long-lived species (Figure 2). Because the *cis*-anionic chromophore displays a characteristic C=C stretching mode at 1655 cm^−1^ and phenol-1 mode at 1623 cm^−1^ based on the static FTIR difference (on minus off) spectra, which are both absent in the spectra of the long-lived species (Figure 2a), the authors concluded that proton transfer is a ground state process while the 9 ps transition time was attributed to the decay of the *trans*-neutral chromophore (likely involving the ultrafast *trans*-*cis* isomerization to result in a *cis*-neutral chromophore) in the excited state. They also monitored the CN_3_H_5_^+^ stretching band of Arg66 (an adjacent protein residue) and the C=O band of the chromophore (HBDI embedded in the protein pocket, Figure 1a) starting from the on state after photoexcitation and showed that hydrogen (H)-bond weakening between the chromophore and local residues is highly related to the much less efficient on-to-off switch (PQY ≈ 3 × 10^−4^) than the off-to-on switch (PQY ≈ 0.3). This work represents an important contribution because it demonstrated that ultrafast vibrational spectroscopy, which is intrinsically sensitive to molecular structural changes, can track the chromophore isomerization processes of RSFPs in real time. Moreover, the capability of TRIR to probe the vibrational bands of surrounding residues demonstrates the importance of protein residues in the chromophore vicinity in determining the photoswitching rate and efficiency of the overall protein. However, the spectral and temporal detection window (~140 cm^−1^ and up to 100 ps, respectively) in this study were rather narrow, which limits efforts in building a more comprehensive model based on the spectral data.

Later in the same year, Lukacs et al. implemented TRIR and investigated a Dronpa mutant, Dronpa-2, because of its high PQYs between the on and off states [56]. They were able to cover a much broader window over 300 cm^−1^ in transient IR spectra and up to a few hundreds of ps after 400 nm photoexcitation, enabling the tracking of more protein modes on the sub-ns timescale. In this study, they discovered that the nearby conserved E144 residue in Dronpa-2 (corresponding to E222 in wtGFP [25,57]), which is H-bonded to the phenolic end of the chromophore through an intervening water molecule and is the potential proton acceptor for the ESPT reaction [33,49,50], does not exhibit a rise of the carbonyl mode at 1710 cm^−1^ and decay of the carboxylate mode at 1560 cm^−1^, both of which are TRIR signatures for the ESPT process in wtGFP. This finding agreed with Warren et al.’s result that the proton transfer is a ground-state thermal process [55]. However, since Lukacs et al. did not observe a blueshift of the C=O mode at ~1690 cm^−1^ that was considered as a signature for *trans*-to-*cis* isomerization, they concluded that the chromophore torsion does not occur on the ps timescale, contradictory to Warren et al. [55]. Instead, they speculated that on the sub-ns timescale (starting from the ps radiationless relaxation), the H-bonding network reorganization in the chromophore vicinity constitutes the dominant process, leading to the formation of a metastable ground state, and the subsequent isomerization would occur on much longer timescales beyond the ns regime. Their explanation of the sub-ns H-bonding network rearrangement between the chromophore and local residues sheds some light on the small kinetic isotope effect (~2) that was observed on the ~4 ps timescale [44], because restructuring of a rather flexible H-bonding network does not need to involve highly directional proton motions from the chromophore and hence reduce the proton transfer rate difference upon deuteration [57,58]. Nevertheless, the exact nature of that metastable ground state remains unclear, which requires further investigations with high-level quantum calculations and spectroscopic techniques with even longer detection time windows (e.g., ms timescale).

**Figure 3:**
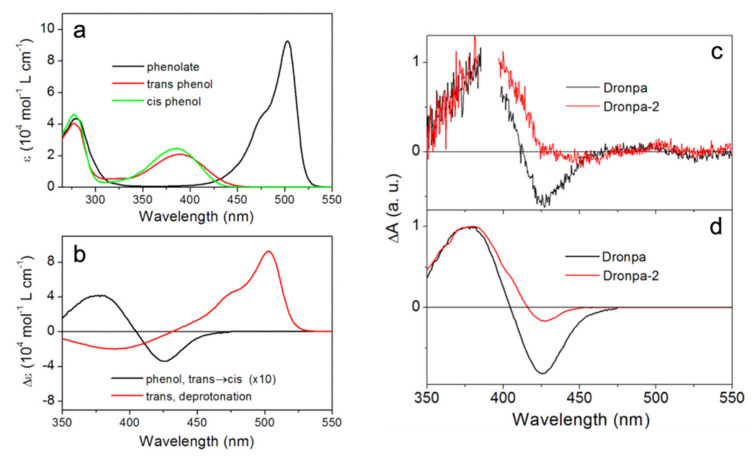
Characteristic electronic features of Dronpa and Dronpa-2. (**a**) Steady-state absorption spectra of Dronpa in the *cis*-phenolate (black), *trans*-phenol (red), and *cis*-phenol (green) forms. (**b**) Difference spectra of the *cis*-phenol minus *trans*-phenol form (black; an isomerization event) and the *trans*-phenolate minus *trans*-phenol form (red; a deprotonation event). (**c**) Decay-associated difference spectra (DADS) of the long-lived (ns) species of Dronpa (black) and Dronpa-2 (red) from global analysis of the transient absorption data. (**d**) The smoothed steady-state difference spectra of the chromophore’s phenol forms (*cis* minus *trans*) in Dronpa (black) and Dronpa-2 (red). Reprinted with permission from Ref. [59]. Copyright 2014 American Chemical Society.

In 2014, Yadav et al. implemented time-resolved UV/visible TA spectroscopy to interrogate the photoactivation pathway of both Dronpa and Dronpa-2 [59]. Compared to the earlier fs-TA work [44], they were able to cover a much broader probe range from 350 to 740 nm, augmented by the anisotropy data. Different to all previous works [44,54,55,56], the unique combination of their fs- and ns-systems made it possible to track the protein evolution from ~100 fs to 1.5 ns and from 5 ns to 10 ms after 388 nm photoexcitation; thus, covering the entire off-to-on switching timescales. The authors identified the distinct electronic absorption spectra of the *cis*-phenolate (anionic form, bright/on state), *cis*-phenol (low-pH-induced neutral form), and *trans*-phenol (photoinduced neutral form, dark/off state) (see Figure 3a). These results were later supported by the resonance and preresonance Raman study of Dronpa in aqueous buffer solutions at various pH values [60], wherein Raman maker bands serve as sensitive reporters for the chromophore configuration in local protein environment [61,62]. Global analysis of their TA spectra up to 1.5 ns yielded four decay-associated difference spectra (DADS) with time constants ranging from sub-ps to tens of ps and a long-lived plateau (exceeding the detection window) (Figure 3c). The long-lived species can be attributed to a ground-state *cis*-phenol intermediate, which exhibits highly similar electronic features to the UV/visible difference spectra of the phenol form (Figure 3c,d), so the authors concluded that the *trans*-to-*cis* isomerization occurs in a few tens of ps, preceding proton transfer. This result was corroborated by their measured small anisotropy (~0.2) of the intermediate species and was also in line with Warren et al.’s conclusion [55]. In addition, they studied the effect of H/D exchange on the retrieved lifetimes. In contrast to Fron et al.’s results [44], they observed no significant slowdown of all the sub-ns processes, indicating the absence of ESPT.

The difference between these experiments was likely due to the fact that, in this study, Yadav et al. covered a much broader spectral and temporal window and performed global analysis that took all transient electronic features into account. This more robust strategy allowed them to eliminate most of the fluctuations generated across a wide spectral region during TA experiments, whereas the isotope effect obtained by Fron et al. was only based on exponential fitting of the TA signal at a single probe wavelength of 420 nm [44]. On the ms timescale, Yadav et al. observed a kinetic isotope effect of 3.5/6.5 at the ~380 and 500 nm probe regions of Dronpa/Dronpa-2, and the time constants of ground-state deprotonation were assigned around 12.5 and 19.2 microseconds (μs) for Dronpa and Dronpa-2, respectively, in H_2_O buffer solutions at pH 8.0. Notably, this work represented the first study tracking the photoactivation process of an RSFP from the start to finish. This unique line of inquiry allowed the authors to draw a comprehensive picture of the Dronpa and Dronpa-2 photoactivation and gain further insights into the turn-on process. This work also provided direct evidence of excited-state isomerization and ground-state proton transfer (GSPT) with the multiexponential kinetic fits mainly attributed to conformational heterogeneity of the off state [32,49]. However, the exact twisting routes of the *trans*-to-*cis* isomerization (e.g., along which dihedral angle of the chromophore, see Figure 1b) and whether the *trans*-neutral chromophore can reach a full *cis* geometry in the excited state were not resolved.

During that same year, Kaucikas et al. implemented TRIR to study Dronpa-2 [63], an investigation that was similar to the aforementioned work by Lukacs et al. [56]. Due to the high PQY of Dronpa-2 during both forward and reverse switching [53], the authors were able to study the photobleaching process in addition to photoactivation. Additionally, this work used a 1 kHz laser system versus the 10 kHz laser system used by Lukacs et al. [56]. Taking advantage of the 1 kHz repetition rate, Kaucikas et al. collected the IR spectra at −100 ps time delay to resolve the 1 ms transient IR features, which closely match the off-minus-on and on-minus-off FTIR difference spectra after 503 nm (on state) and 400 nm (off state) excitation, indicating that chromophore isomerization and proton transfer are completed on this timescale. This result is consistent with the aforementioned findings by Warren et al. about thermal proton transfer [55] and Yadav et al. that ground-state deprotonation timescale is ~19.2 μs for the photoactivation of Dronpa-2 [59].

**Figure 4:**
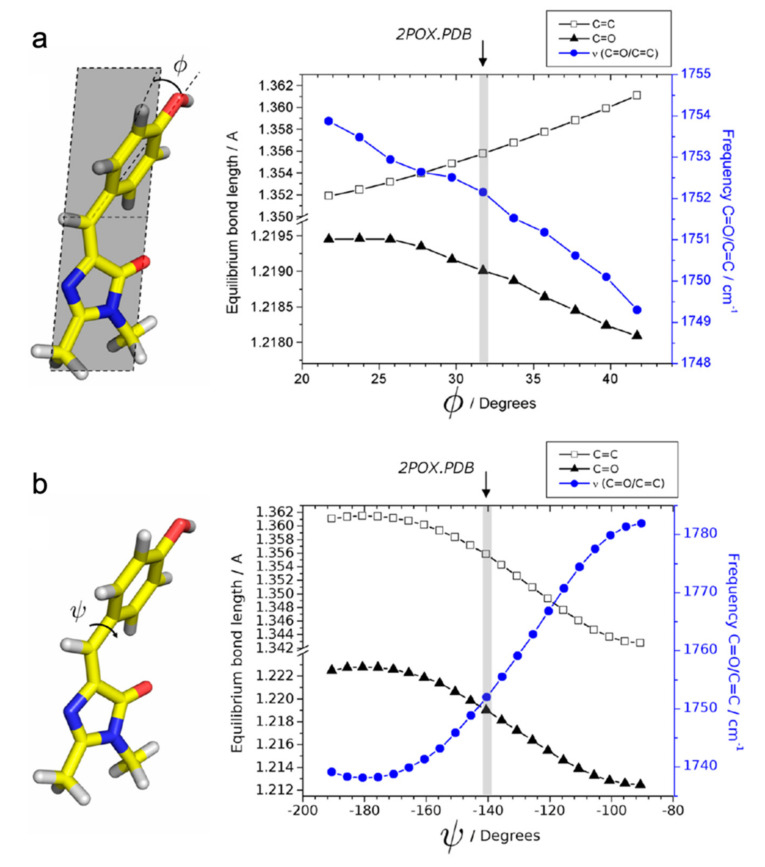
Dihedral-angle dependence of the calculated vibrational frequency and bond lengths of the neutral HBDI chromophore. (**a**) Dependence on the *Φ* angle (phenol ring out-of-plane bending). (**b**) Dependence on the *ψ* angle (phenol ring torsion). Angles (*Φ* = 32°, *ψ* = −140°) corresponding to the off-state Dronpa crystal structure (PDB ID: 2POX) [32] are denoted by the gray vertical bars. Reprinted with permission from Ref. [63]. Copyright 2014 American Chemical Society.

For the off-to-on activation, the authors retrieved three time constants of 0.6 ps, 14 ps, and 17 ns from global analysis of their TRIR spectra. The multi-exponential components may arise from the inhomogeneous ground state with structural disorder [49]. Kaucikas et al. attributed the first two lifetimes to isomerization, consistent with the 9 ps component reported by Warren et al. [55], but against the assignment by Lukacs et al. [56]. To rationalize the absence of an expected C=O mode frequency blueshift, Kaucikas et al. argued that the C=O group on the chromophore imidazolinone ring could H-bond with Arg66 to decrease the observed vibrational frequency. They also argued that a full isomerization may not be completed on this timescale. Based on their density functional theory (DFT) calculations of the protonated chromophore as the off-state dominant species, the phenol-ring out-of-plane bending from 32° to 20° only causes a very small (~2 cm^−1^) blueshift (Figure 4a) while the phenol ring torsion from −140° to −90° leads to a much more dramatic (~30 cm^−1^) blueshift (Figure 4b). It is possible that the chromophore mainly undergoes the out-of-plane bending motion on the ps timescale, and generates the primary photoproduct as the *cis*-neutral chromophore with other structural rearrangements on the ns timescale [63]. This work thus raised a very important concept that the chromophore isomerization should not be simply treated as a one-step or one-coordinate process, which is intimately governed by the multidimensional potential energy surfaces from ground to excited states of such condensed-phase molecular systems [39,64]. Instead, the rotation around various dihedral angles and their temporal sequences should be taken into consideration for a more complete and detailed picture of the chromophore conformational twisting routes in RSFPs (Figure 1b).

**Figure 5:**
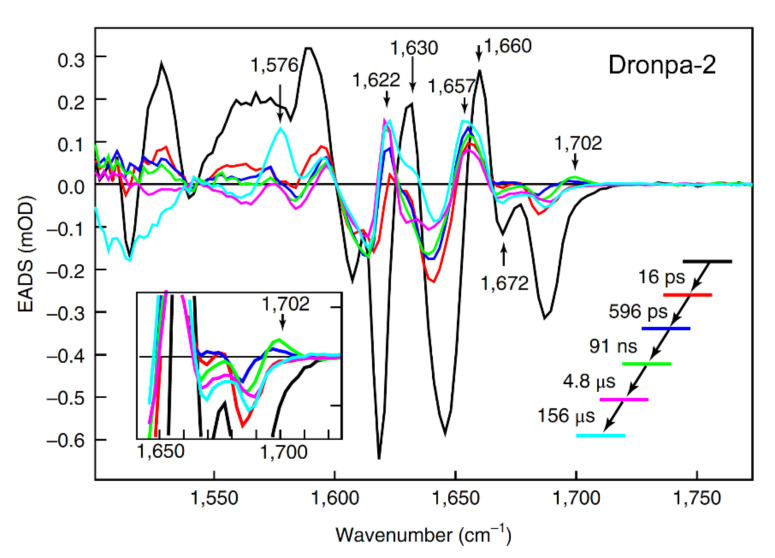
Global analysis of TRIR spectra of Dronpa-2 in D_2_O solution. A sequential model yields five evolution-associated difference spectra (EADS) with key vibrational modes denoted by vertical arrows. Reprinted with permission from Ref. [46]. Copyright 2018 Springer Nature.

In 2018, Laptenok et al. reported the complete photocycle for the off-to-on activation process of Dronpa-2 in D_2_O using fs-to-ms TRIR spectroscopy with isotope labeling to aid vibrational mode assignments [46]. Global analysis of their TRIR spectra with a sequential model yielded five time constants: 16 ps, 596 ps, 91 ns, 4.8 μs, and 156 μs (Figure 5). The major finding of this study was the rise of a 1702 cm^−1^ mode, which is blue-shifted from the 1689 cm^−1^ C=O mode, with a 91 ns time constant: they attributed this process to *trans*-to-*cis* isomerization of the neutral chromophore in the ground state. Although a previous calculation showed that the ground state isomerization barrier is very high (~2.1 eV) [63], the authors argued that the protein environment can significantly reduce the barrier and make the 91 ns isomerization step possible in S_0_. This assignment was at odds with the observation of Yadav et al. [59], Warren et al. [55], and Kaucikas et al. [63], all assigning the tens of ps time constants (similar to the 16 ps retrieved by Laptenok et al. [46]) to isomerization. The authors recognized that the intermediate formed after ~16 ps should have a different electronic structure from the *trans*-chromophore, but its exact geometry could not be determined from their TRIR study, which may require high-level quantum calculations or femtosecond crystallography. The 596 ps and 4.8 μs components were assigned to stabilization of the intermediates while the 156 μs step in D_2_O was attributed to GSPT, which is close to the 124 μs observed by Yadav et al. [59], before reaching the final *cis*-anionic form in a structurally reorganized protein pocket with a significantly modified H-bonding network.

Overall, regarding the off-to-on photoactivation mechanisms of Dronpa and Dronpa-2, multiple ultrafast techniques including time-resolved fluorescence, fs/ns-TA and TRIR, which cover various spectral and temporal ranges, have been implemented. The current consensus about this process is that the chromophore *trans*-*cis* isomerization precedes GSPT. However, whether or not the isomerization occurs in the excited or ground state remains debated while the retrieved time constants and associated interpretations are still inconsistent across different techniques. In addition, even the reported PQYs and FQYs of Dronpa and Dronpa-2 have notable variations in literature [21,22,53,59,63]. Furthermore, all studies could not identify the exact chromophore twisting structures or coordinates in the excited or ground state. Therefore, novel ultrafast techniques, such as femtosecond stimulated Raman spectroscopy (FSRS) [39,57,62,65] and the time-resolved serial femtosecond crystallography [45], as well as high-level quantum calculations and molecular dynamics simulations [38,51] are needed to further elucidate the detailed switching pathways inside RSFPs.

### 2.2. RsEGFP2

RsEGFP2 is an engineered descendent of the well-known wtGFP, which was discovered from the Pacific Northwest jellyfish *Aequorea victoria* (Hydrozoa) in 1962 [66] and adopts a Ser65-Tyr66-Gly67 (SYG) chromophore [2,67]. To improve the brightness of wtGFP, two point mutations (S65T and F64L) were carried out to yield an enhanced GFP (EGFP) in 1996 with 35-fold more fluorescence intensity and an FQY of ~0.60 [68]. In 2011, Grotjohann et al. developed an EGFP-based monomeric RSFP named reversibly switchable EGFP (rsEGFP) by introducing additional mutations (Q69L, V150A, V163S, S205N, and A206K) to EGFP via site-directed and error-prone mutagenesis [8]. RsEGFP was able to undergo ~1100 cycles of photoswitching before its fluorescence intensity decays by 50% and the switching rate is more than 10-fold faster than Dronpa (see Section 2.1 above). However, rsEGFP was still rather slow when implemented in the subdiffraction-resolution RESOLFT microscopy that employs the reversible saturable optical fluorescence transitions between two (on and off) states in a spatially targeted manner. In 2012, Grotjohann et al. continued to develop rsEGFP2 with only four mutations (T65A, Q69L, V163S, and A206K) based on EGFP [69]. RsEGFP2 adopts an Ala65-Tyr66-Gly67 (AYG) chromophore and was able to record images in RESOLFT 25–250 times faster than rsEGFP. At pH 7.5, rsEGFP2 rests in its bright state with an excitation maximum at 478 nm and a fluorescence peak around 503 nm. A 480 nm light can induce fluorescence from rsEGFP2 with FQY ≈ 0.30 but can also bleach it (yet the fluorescence was halved not before ~2100 cycles of photoswitching [69]), and 400 or 405 nm irradiation activates it again. The on-to-off and off-to-on PQYs of rsEGFP2 are 1.65 × 10^−2^ and 0.33, respectively [70], later reported as 0.04 and 0.40 [45] or 0.01 and 0.18 [71]. Based on its crystal structure in the on and off states with specific H-bonding patterns, the photoswitching mainly involves the *cis*-*trans* isomerization and proton transfer, similar to other negative RSFPs [11]. Notably, since both one-bond flip and hula twist can lead to the formation of a *trans*-chromophore, a recent study by Chang et al. revealed that for rsEGFP2, the protein packing in the crystal lattice is a determining factor for the photoswitching pathways: a loose and tight packing configuration leads to the one-bond flip and hula twist, respectively [72].

**Figure 6:**
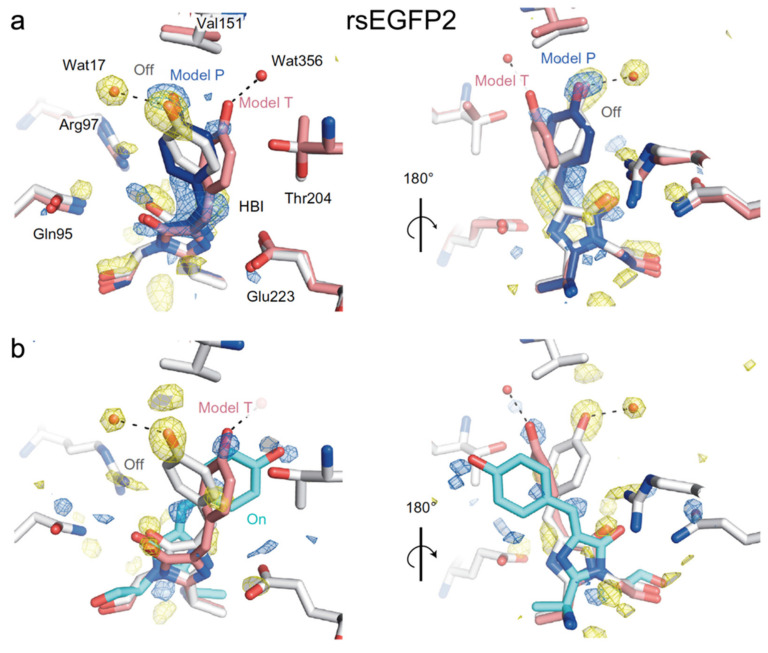
Local structure of rsEGFP2 in the first singlet electronic excited state modeled from difference Fourier electron-density maps of SFX data. (**a**) Comparison of Model P (blue), Model T (pink), and the off state (gray) against SFX map at 1 ps after photoexcitation (PDB ID: 5O8B) [45]. (**b**) Comparison of Model T (red) with the off (gray) and on (cyan; PDB ID: 5O89) states against SFX map at 3 ps after photoexcitation. Note that in model T, the twisted chromophore features perpendicular phenol and imidazolinone rings. Key surrounding residues are shown with the H-bond denoted by dashed lines. Reprinted with permission from Ref. [45]. Copyright 2017 Macmillan Publishers Limited, part of Springer Nature.

In 2018, Coquelle et al. performed the first ultrafast study on rsEGFP2 with fs-TA and time-resolved serial femtosecond crystallography (TR-SFX), focusing on its early time (sub-ns) structural change [45]. Guided by the fs-TA spectra of rsEGFP2 in aqueous solution (pH/pD 8) which exhibit a strong signal at ~1 ps and a significant decay at 3 ps, they collected the TR-SFX data at the two delay time points after 400 nm photoexcitation. They resolved two conformers (model P (planar) and model T (twisted)) at 1 ps (Figure 6a), but only model T and the *cis* structure were clearly observed at 3 ps (Figure 6b). This key result indicated that the *trans*-*cis* isomerization has already occurred by 3 ps following electronic excitation. The structure of Model P closely resembles the off state (Figure 6a) while the geometry of model T is in between the *cis* and *trans* geometries (Figure 6b). The existence of two conformers was supported by their excited-state quantum mechanics/molecular mechanics (QM/MM) and classical MD simulations. Because twisting of the *trans*-chromophore to form model T, which is the precursor of the *cis*-form, involves both dihedral angles, they attributed the photoactivation of rsEGFP2 to a hula-twist mechanism. In the resolved crystal structure of rsEGFP2 at 3 ps (Figure 6b), V151 sterically hinders further motions in model T at the phenol side, so the authors performed a targeted mutation (V151A) to reduce the hindrance: the off-to-on PQY of the new rsEGFP2 mutant almost doubles when compared to the parent protein based on their PQY measurements. However, they later discovered that the measurement was incorrect and there is no significant difference between the PQYs of rsEGFP2 and rsEGFP2-V151A [73]. This result showcases the complexities in point mutations which may also induce additional changes to the chromophore’s surrounding residues that can lead to an outcome deviated from expectations. The study by Coquelle et al. offered a good example of utilizing ultrafast spectroscopic techniques, in combination with quantum and classical calculations to retrieve the underlying molecular mechanisms. However, unresolved questions linger from this study [45]. For example, the fs-TA spectra reveal three excited states: a Franck–Condon state with a 90 fs decay and two additional intermediate states with 0.9 and 3.7 ps decay time constants. The nearly planar Model P was attributed to one state whereas the twisted Model T was attributed to neither state because its calculated transition dipole moment is close to zero. The exact nature of the other state was unclear, while the SE band dynamics near the deprotonated chromophore fluorescence peak (~503 nm) could be further analyzed. Moreover, the study offered no information about the photoactivation process of rsEGFP2 on longer timescales (e.g., ns to ms).

**Figure 7:**
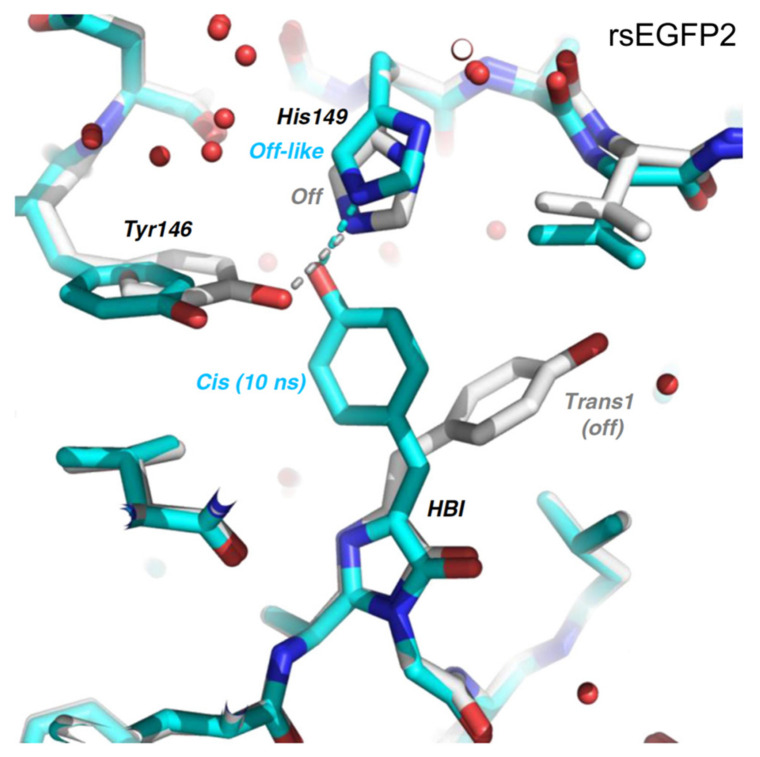
The 10 ns structure of rsEGFP2 during photoactivation. The *cis*-chromophore in the 10 ns intermediate (cyan; PDB ID: 6T3A) is overlaid with the *trans*-chromophore in the off state (gray, 65% occupancy major conformer), accompanied by a notable H-bonding partner change of His149 in off state (to Tyr146) versus 10 ns intermediate (to chromophore), denoted by the color-coded dashed lines. Reprinted with permission from Ref. [74]. Copyright 2020 The Authors.

Subsequently in 2020, Woodhouse et al. studied rsEGFP2 again with TA and TR-SFX but focused on the longer timescale beyond ps [74]. They collected the TR-SFX data of rsEGFP2 at 10 ns and observed that the chromophore is already in the *cis* geometry on this timescale, the same as the “target” on state but prior to chromophore deprotonation on the μs-to-ms timescale (see above). However, the side chain of the adjacent residue His149 is still at an off-like position (Figure 7); note these SFX data were modeled with ~10% remaining on-state population and other inhomogeneous conformational species (including a key residue His149 within H-bonding distance of the chromophore phenolic end). Global analysis of the fs-TA data of rsEGFP2 in its off state upon photoexcitation in aqueous buffer solution (pH 8) from 40 ps to 2 ns retrieved a single lifetime of 87 ps, which was attributed to the reorganization of the protein pocket to accommodate the *cis*-chromophore. Global analysis of the ns-TA data from 100 ns to 10 ms yielded three time constants of 5.57, 36.1, and 825 μs, with the latter two components exhibiting H/D kinetic isotope effect of ~2.5 that corresponds to GSPT steps, reminiscent of similar processes in Dronpa-2 [46,59]. The 5.57 μs component could arise from the reorganization of His149 or the other surrounding residues. Structural information about the nature of the 87 ps and 5.57 μs components was lacking in this study.

To reveal the unresolved intermediate states on the ps and μs timescale from the previous two studies [45,74], Uriarte et al. recently implemented fs-to-ms time-resolved multiple-probe infrared spectroscopy (TRMPS-IR) with the Savitzky–Golay derivative filter to study rsEGFP2 in solution [71]. Global analysis of the IR spectra from 1 ps to 2 ns revealed three DADS with time constants of 0.7, 5.5, and 117 ps, similar to the 0.9, 3.7, and 87 ps from earlier reports [45,74]. The 0.7 ps DADS is characterized by a decay of the 1681 cm^−1^ C=O stretch band and rise of a positive 1686 cm^−1^ band. This characteristic blueshift from 1681 to 1686 cm^−1^ is a signature of *trans*-to-*cis* isomerization that indicates the formation of a *cis*-photoproduct precursor on this sub-ps timescale (i.e., a highly efficient ring twist). The result is consistent with the observation of the Model T chromophore at 1 ps from the TR-SFX experiment [45]. During the 5.5 ps transition time, only the recovery of vibrational bands from the *trans*-neutral chromophore was observed; thus, it was attributed to the relaxation of a hot planar *trans*-chromophore back to the original off state. On the 117 ps timescale, a further blueshift of the 1686 cm^−1^ mode to 1691 cm^−1^ was detected, suggesting that the chromophore continues to twist to its final *cis*-neutral state, similar to the 10 ns structure (Figure 7) observed in TR-SFX [74]. On longer timescales from 1 ns to 900 μs, four DADS were retrieved, including a new 42 ns component that had not been resolved from previous TA data. This component leads to a significant change in the 1651 cm^−1^ amide band from protein residues close to the chromophore, likely the His194 side chain (see Figure 7). No significant change in the TRIR spectra occurs on the 2.2 μs timescale, likely associated with residues distant from the chromophore, such as the β-barrel relaxation (Figure 1a). Similar to the 10 ns TR-SFX study [74], Uriarte et al. also observed two time constants of ~67 μs and 2 ms which were attributed to GSPT due to the appearance of a phenolate vibrational band at 1491 cm^−1^ [71]. Overall, by following the detailed dynamic patterns of carefully filtered vibrational marker bands, this most recent study supported the major conclusions from the TR-SFX investigations [45,74] and also discussed the previously hidden 42 ns process. Their results can be further verified by high-level excited/ground state quantum calculations and MD simulations (e.g., for accurate IR mode assignments) as well as targeted mutations of the protein.

Although the first ultrafast study of rsEGFP2 (hydrozoan origin) appeared more than 10 years later than Dronpa (anthozoan origin), a clearer photoactivation picture has been drawn for rsEGFP2. The timescale and sequence of isomerization and proton transfer are relatively consistent across various time-resolved electronic (TA) and vibrational (IR) spectroscopies. More importantly, the excited-state intermediate structures that govern the photoisomerization efficiency and rate have been solved, as well as the ground-state structure during further chromophore and protein relaxation. The experimental results have been correlated with theoretical calculations. However, only three crystal structures of rsEGFP2 at 1, 3 ps [45] and 10 ns [74] were solved, which limits painting a more comprehensive photoinduced reaction portrait. Investigations on the finer chromophore and surrounding pocket structures between ps and ns as well as beyond the μs timescale, also for RSFPs in physiological environments such as aqueous buffer solution under irradiation, can fill the missing links during off-to-on switching and offer additional important insights into the photoactivation pathways of RSFPs in living systems.

### 2.3. IrisFP

IrisFP was engineered from EosFP, which is a stony-coral-derived (anthozoan) green-to-red photoconvertible tetrameric fluorescent protein with a His62-Tyr63-Gly64 (HYG) chromophore [75], through a single-site F173S mutation [76]. Comparing to Dronpa and rsEGFP2, IrisFP inherited the photoconversion capability from its ancestor and it can also photoswitch reversibly within its green and red forms. The unique multi-photochromic behavior of IrisFP makes it a promising tool for advanced bioimaging applications. In aqueous buffer solution at pH 9, IrisFP exists in its green form with a major absorption band at ~488 nm and a minor band around ~390 nm. Excitation at 488 nm yields strong fluorescence at 516 nm with an FQY of 0.43. The 488 nm light can also convert the protein to an off state that absorbs at ~390 nm, with an on-to-off PQY of 0.014. A 405 nm light has the dual function to efficiently turn the protein back on (PQY ≈ 0.5) and convert the protein to red form (photoconversion QY ≈ 0.0018). The red species absorbs strongly at ~551 nm and emits at 580 nm with an FQY of 0.47, comparable to the green form. The red form can also be switched between its on and off states by the 532 and 440 nm light separately, with a much-reduced PQY (on-to-off ~0.0020, off-to-on ~0.047) when compared to the green species. Note that the off-to-on PQY is consistently much larger than the on-to-off PQY. Confirmed by crystal structures of the green and red forms, the photoswitching pathway involves *cis*-*trans* isomerization of the chromophores in both states [76].

**Figure 8:**
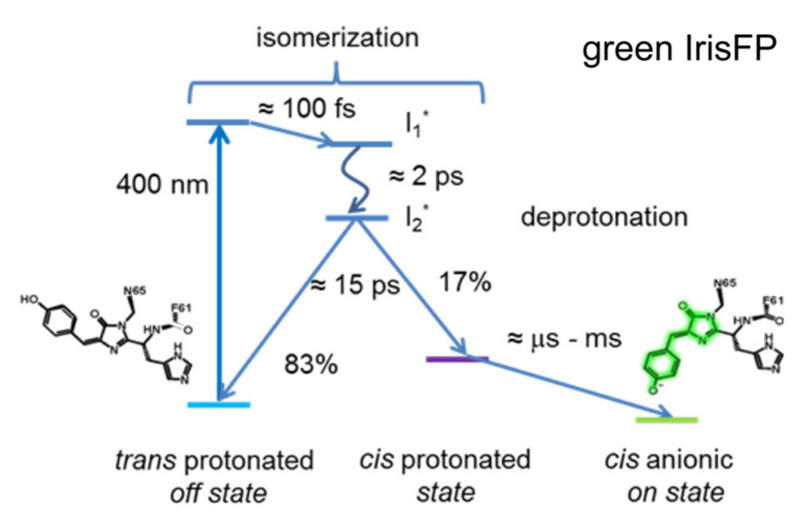
Photoactivation mechanism for green IrisFP in aqueous solution. Following 400 nm fs-laser excitation, the protein chromophore undergoes the excited-state *trans*-to-*cis* isomerization on the ps timescale while the deprotonation events occur on the μs-to-ms timescales. The asterisk indicates the electronic excited state for the pertinent chromophore species. Chemical structures of the chromophores in the off (dark) and on (green) states are depicted. Reprinted with permission from Ref. [77]. Copyright 2016 American Chemical Society.

To unravel the detailed photoactivation mechanism of IrisFP in the green state, Colletier et al. implemented time-resolved TA in 2016 [77]. They utilized a similar strategy as Yadav et al. in studying Dronpa and Dronpa-2 [59], and used fs-TA with a 400 nm pump to cover from 100 fs to 1 ns time regime and ns-TA (laser flash-photolysis) with a 410 nm pump to cover from 10 ns to 10 ms for IrisFP in pH 7 solution. Although the 400/410 nm light can also induce photoconversion, the photoactivation QY (0.5) is more than ~270-fold higher than the photoconversion QY (0.0018) [76]. Therefore, it is reasonable to consider that photoconversion contributes an extremely small portion to the observed fs-TA spectra and does not hinder the study of photoactivation of green IrisFP. Within the sub-ns window, global analysis yielded three time constants of ~100 fs, 2.02 ps, and 15.3 ps (Figure 8). The 100 fs component was assigned to the initial intramolecular vibrational relaxation of the chromophore, while the ~2 and 15 ps components were attributed to the lifetimes of two excited states (I_1_* and I_2_*) with a sequential relation, i.e., I_1_* proceeds to I_2_* with 2 ps time constant, then I_2_* relaxes back to the original ground state and the *cis*-protonated state with 15 ps time constant (Figure 8). In this case, the structure of I_2_* is crucial which determines the efficiency and yield of the *trans*-to-*cis* isomerization. On longer timescales (ns-ms), fitting the 490 nm absorption region yielded three exponential components in H_2_O solution: 21 μs (33%), 227 μs (21%), and 2.1 ms (46% amplitude weight), all of which exhibit H/D kinetic isotope effects ranging from ~2.5 to 12, indicating that proton dissociation on these timescales may involve multiple steps or populations. This phenomenon was also observed from the TA and TRIR data of rsEGFP2 [71,74].

Since the structures of I_1_* and I_2_* were unknown from the TA data, to characterize these important early-time excited-state intermediates in possible correlation with the *trans*-*cis* isomerization of the chromophore, the authors examined the feasibility of using SFX and an X-ray free electron laser (XFEL, 5–50 fs pulse duration) to obtain the IrisFP on-state structure. The structure was then successfully modeled based on the SFX data, also checking the mineral-grease-embedding effect on protein microcrystal structure to be minor, which closely resembles the structure retrieved from traditional (synchrotron) crystallography of a flash-cooled control crystal sample at 100 K. However, the SFX structure displays two conformers of Arg66 that can stabilize the *cis*-chromophore through H-bonds [77], and this bimodal structure may represent a more realistic case since the experiment was performed at room temperature. Notably, the authors built upon their earlier success of using SFX to solve the on-state structure of IrisFP and continued to study the photoactivation routes of rsEGFP2 in 2018 [45] and 2020 [74], wherein they reported some representative structures of intermediate states in the excited and unrelaxed ground states of a prototypical negative RSFP as summarized above.

On a related note, our lab recently implemented fs-TA and ground-state FSRS techniques to study a green-to-red photoconvertible fluorescent protein (PcFP) called the least-evolved ancestor (LEA) to retain the green phenotype with photoconversion capability [78]. Like IrisFP, this coral-derived LEA is also a tetrameric FP and can be switched between a bright and dark state in the green form with 505 nm (on-to-off) and 400 nm (off-to-on) light, respectively. Interestingly, unlike IrisFP and other RSFPs in which the photoinduced off-state species can automatically return to the bright state after dark adaptation, the light-driven off-state species in LEA is semi-trapped and cannot be turned on without light irritation [78]. This is likely due to the unique protein pocket and interactions between the LEA chromophore and local surrounding protein residues, which can undergo the photoinduced rearrangement in concert [79]. Ultrafast spectroscopic studies (fs-TA in the electronic domain, FSRS in the vibrational domain), quantum calculations, and MD simulations are ongoing in our group to characterize this intriguing semi-trapped species (still capable of photoconversion to generate red emission) that may provide the useful expanded frameworks to derive more general principles for photoswitching in competition with photoconversion.

### 2.4. Other Negative RSFPs

Many other negative RSFPs have been engineered from teal color to the less phototoxic redder regions, such as mTFP0.7 [33,80,81], also the monomeric rsCherryRev [82], rsTagRFP (turned on by 440 nm light and off by 567 nm light, much brighter than rsCherryRev) [83], and three rsFusionRed variants (multiple irradiation wavelengths at 405/488/510 nm can be used to switch on the protein, with 590 nm light capable of switching off the protein; faster off-switching kinetics than rsTagRFP and rsCherryRev1.4) [18], by varying the chromophore composition and mutagenesis of local residues. Note that these redder proteins (than Dronpa or rsEGFP2) display some intriguing properties from multiple switching locations [18] to an unusual planar *trans*-chromophore that is nonfluorescent [83]. However, there have not been ultrafast studies performed on them, which represents a wonderful opportunity for researchers to implement advanced ultrafast techniques to examine these fascinating proteins. The systematic investigation and comparison of multiple negative RSFPs with contrasting properties would significantly benefit their future development in a synergistic manner. These related studies would allow us to evaluate whether the retrieved ultrafast mechanisms (see above, particularly regarding the excited-state chromophore twist on the ps timescale) can be universally applied to negative RSFPs. It is expected that with a different chromophore and local protein residues, the timescales of isomerization and proton transfer would vary to different extents. However, some essential questions warrant special attention. For example, does *trans*-*cis* isomerization always occur before proton transfer? Can we always see an excited-state structure that is in-between the *cis* and *trans* geometries for all RSFPs? Which local environment favors the one-bond rotation or hula twist and are these processes mutually exclusive of each other? What is the major determinant for the protonation and conformational states of the chromophore with various usual or unusual combinations of *cis*-/*trans*-/neutral/anionic/planar/nonplanar descriptors? The growing pertinent comprehensive experimental findings with more advanced spectroscopic toolsets are expected to help us to further understand the photoactivation mechanism from the bottom up and efficiently guide the future research directions and engineering strategies to develop next-generation negative RSFPs.

## 3. Positive RSFPs

**Figure 9:**
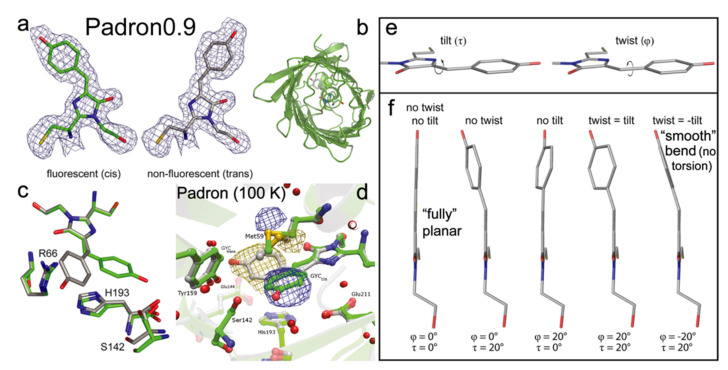
Molecular basis for the positive-switching Padron0.9 and Padron. (**a**) The CYG chromophore of Padron0.9 was determined to be in fluorescent (*cis*-) and nonfluorescent (*trans*-) states from X-ray crystal structures [35]. (**b**) Aerial view of the protein β-barrel (green) with the embedded chromophore in the center (green, on state; gray, off state). (**c**) No significant rearrangements of the surrounding residues (e.g., R66, S142, H193) occur in the on (green) and off (gray) states despite the chromophore isomerization. (**d**) Padron in low-temperature crystals after 532 nm irradiation at 100 K displays the chromophore isomerization from *trans*- (off, gray) to *cis*- (on, green) states with some key residues (e.g., S142, E144, Y159, H193, E211) in the protein matrix showing small rearrangements. (**e**) Illustrative modulus of the sum of two bridge dihedral angles (τ, close to I ring, tilt; ϕ, close to P ring, twist) as a measure for two chromophoric ring torsional motions. (**f**) A smooth bend is only achievable when τ and ϕ values have the same magnitude but opposite signs. Adapted with permission from Ref. [35]. Copyright 2010 The American Society for Biochemistry and Molecular Biology, Inc. Adapted with permission from Ref. [84]. Copyright 2011 American Chemical Society.

Padron is a monomeric positive RSFP, engineered from Dronpa with eight mutations (T59M, V60A, N94I, P141L, G155S, V157G, M159Y, F190S) [22]. The V157G and M159T mutations are the most critical in introducing the positive switching mode. Pardon adopts the same CYG chromophore as Dronpa. In the on state, Padron exhibits a minor absorption peak around 395 nm from the protonated chromophore, and a major peak at 503 nm due to the deprotonated chromophore. The ~503 nm light can generate strong fluorescence with an FQY of 0.64 and it can also turn the off species on. The ~400 nm light can “bleach” the on state and yield a dark protein (FQY~7 × 10^−3^) with a single dominant absorption peak at 500 nm [22]. Note that Padron adopts the dark state at thermal equilibrium. The switching phenomena are opposite to Dronpa in which the bluer light turns the protein on, and the redder light switches it off [54]. The peculiarity of Padron lies in a proposed rapid equilibrium between the *cis*-neutral (protonated, 396 nm absorption peak) and *cis*-anionic (deprotonated, 503 nm absorption peak) chromophores, which can be switched off by UV (~400 nm) light [22]. Notably in this case, the resultant *trans*-anionic chromophore state is nonfluorescent (Figure 9a), but whether the protonation or conformational state plays a more dominant role remains debatable (Figure 1b).

To investigate the unique photoswitching behavior of Padron, Brakemann et al. engineered a variant called Padron0.9 (Figure 9b), which differs by two mutations (Y116C and K198I) from Padron and has a slightly increased tendency to dimerize [35]. This property allowed them to crystalize Pardon0.9 (at pH 6.6 in the dark) and solved its structures in the on and off states. The 488 and 405 nm photoswitching was revealed to involve the *cis* (on)–*trans* (off) isomerization, similar to Dronpa. In line with the multidimensional reaction coordinate and complexity of condensed-phase systems, the authors proposed the modulus of the sum of P- and I-ring bridge dihedral angles (tilt and twist in Figure 9e,f; the smaller values or the more smoothly bent configurations, the higher fluorescence) of the chromophore, along with the protein pocket rigidity, as two key factors for the GFP-like chromophore to be fluorescent [35].

During the on-to-off switch, the p*K*_a_ of the chromophore is dropped by ~1.5 from 6.0 (on state) to 4.5 (off state) based on the titration experiments, in line with their MD simulation results. Therefore, the *trans*-species favor the deprotonated form that absorbs at ~500 nm while the *cis*-chromophore tends to have both forms at physiological pH. The authors also identified a nearby key residue, His193, which is strongly coupled with the chromophore via π-π stacking with its phenol ring (Figure 9c). The imidazolinone ring of His193 needs to be protonated to yield the correct p*K*_a_ shift, indicating that the protonation state of nearby residues can significantly affect the chromophore acidity. The crystallography results also show that despite the large twist/torsion of the chromophore during photoswitching events, the positions of surrounding residues hardly change, in sharp contrast to negative RSFPs (Section 2) [11,45,74]. This finding was later confirmed by Regis Faro et al., who obtained the crystal structure of Padron at low temperature (100 K) during its off-to-on photoswitching [84]. They showed that the photoactivation involves the *trans*-anionic (off) form and *cis*-neutral (nonfluorescent)/*cis*-anionic (on, fluorescent) forms as the start and end structures along with two intermediates states in the middle, upon increasing the temperature from 100 to ~180 K to eventually reach a protonation equilibrium. They also found during the switching only Met59 shows a clear reorientation while other residues (mostly on the other side of the chromophore, see the lower half of the pocket in Figure 9d) exhibit little displacement, which suggests that unlike Dronpa or rsEGFP2, the chromophore in Pardon has much less interaction with the surrounding residues throughout the switching processes; thus, leading to very different excited-state potential energy surfaces and the associated molecular dynamics. In essence, the chromophore *trans*-to-*cis* isomerization is decoupled from the protonation change at the phenolic hydroxy end, enabling the fluorescence photoactivation of Padron even at cryotemperatures [84].

The off-to-on and on-to-off PQYs of Padron are 0.003 and 0.04, respectively [85]. For a few Padron mutants such as Padron0.9, Padron2, and Kohinoor, the photoactivation/bleaching PQYs are 3 × 10^−4^/0.02, 5 × 10^−3^/0.115, and 0.02 (later updated: 0.015)/0.15 (later updated: 0.088), respectively [85,86,87]. Consequently for positive RSFPs, the on-to-off PQY is generally much higher than the off-to-on PQY, exactly opposite to negative RSFPs [11]. Notably, the off-to-on PQYs of negative RSFPs are often more than 30%: 0.37 for Dronpa [21], 0.33 for rsEGFP2 [70], and 0.50 for IrisFP [76], which have enabled the feasibility of utilizing ultrafast techniques to study the photoswitched species. In contrast, the on-to-off PQYs of positive RSFPs are generally below 15%. For Padron, the value is only 4%, which poses a significant challenge to investigate it with ultrafast techniques since most of the observed signals are likely not due to the photoswitching-off pathway, especially for the ultrafast electronic spectra with broad and overlapping peaks [39].

In 2013, Fron et al. performed the first ultrafast study on Padron with fs fluorescence up-conversion, fs-TA, and ps time-correlated single photon counting (TCSPC) techniques [88]. Comparing the fluorescence dynamics of on and off states following 495 nm photoexcitation to induce the off-to-on transition, they identified a time constant of 5.2 ps, which shows a large amplitude in the off state and was attributed to the *trans*-to-*cis* isomerization, leading to a hot ground state or a perpendicular excited state intermediate (turning on fluorescence in this case). However, this assignment may be inaccurate since the off-to-on PQY is only 0.3% [85], so the 5.2 ps component with a notable contribution to the decay process is unlikely due to isomerization. This point was also raised in a later study on Dronpa0.9 [86]. Additionally, the existence of this time constant in both the photoexcited *cis*- and *trans*-anionic forms may further refute this assignment. Moreover, the fs laser at 395 nm the authors used to preserve the sample in the off state may be insufficient due to the low PQY and sample flow [88]. For the on-to-off switching upon 395 nm excitation, the authors revealed a 14.5 ps component by studying a mixture of on and off states and a relatively pure on state, and this time constant was attributed to *cis*-to-*trans* isomerization (turning off fluorescence), which is likely subject to similar issues as mentioned above. Notably for the on-to-off transition of Padron, the authors identified a 1 ps time constant attributable to ESPT (*cis*-neutral to *cis*-anionic in S_1_) since the correlated decay of the ~450 nm SE band and rise of the ~520 nm SE band were observed, corresponding to the decrease in the protonated form and increase in the deprotonated form as reported for other GFP systems [62,89].

In 2015, Walter et al. studied the excited state dynamics of Padron0.9 in its on and off states with fs-TA [86], noting that its off-to-on PQY is even lower (0.03%) than Padron (0.3%). The ground-state titration experiments revealed that the chromophore adopts several protonation states, affected by the protonation states of two nearby residues. This finding agrees with previous crystallography and MD simulation results that local residues can affect the chromophore p*K*_a_ [35]. Due to this reason, the authors systematically designed fs-TA measurements on Padron0.9 *cis*- and *trans*-forms at pH 10 solution with 505 nm excitation (but with 495 and then 400 nm LEDs to track different states) and at pH 4 solution with 387 nm excitation (tracking dynamics of the *cis*-neutral species with the adjacent residues all protonated), as well as in the on state at pH 10 solution with 387 nm excitation (tracking dynamics of the *cis*-neutral species). They discovered that the *trans*-anionic form relaxes back down to a hot ground state efficiently through several intermediate states on the sub-10 ps timescale. The initial excited-state process of the *cis*-neutral chromophore involves a competition between internal conversion and ESPT on the 1.6 ps timescale. Target analysis of the TA spectra revealed an ESPT time constant of 1.7 ps in H_2_O, followed by formation of the fluorescent states (~1.2 ns lifetime) with two discernible *cis*-anionic subpopulations. The ESPT assignment was confirmed by the large isotope effect (5.2 for ESPT and 3.5 for GSPT) upon H/D exchange and is consistent with the 2013 study by Fron et al. on Padron [88], implying that the first step of photoinduced *cis*-to-*trans* isomerization may involve ultrafast chromophore deprotonation in the excited state, facilitated by the local protein environment.

Overall, there has been a limited number of ultrafast studies on positive RSFPs and detailed information is still lacking for the chromophore isomerization process, likely due to very small PQYs in both photoswitching directions as well as multiple interchanging species with similar absorption profiles or low FQY values. Therefore, the development of positive RSFPs with larger PQYs and faster ensemble switching would significantly benefit future spectroscopic characterization of such protein systems. For example, the on-to-off PQY of a recently engineered Pardon2 is around 12% [87], which may be a good candidate for ultrafast studies. Another unresolved question for positive RSFP is the structure of its native resting state without illumination. Because all the protein structures so far were solved in the off and on states after specific light irradiation, whether the native state adopts *cis*- or *trans*-structures remains unclear. Since Pardon and most of its derivatives rest in a dark state, it is likely that they adopt an overall *trans*-structure [35,84]. It would be highly beneficial to resolve its structure in situ since it is the starting point of any photoswitching process and can offer valuable information regarding the true “onset” of such functional proteins.

## 4. Decoupled RSFPs

**Figure 10:**
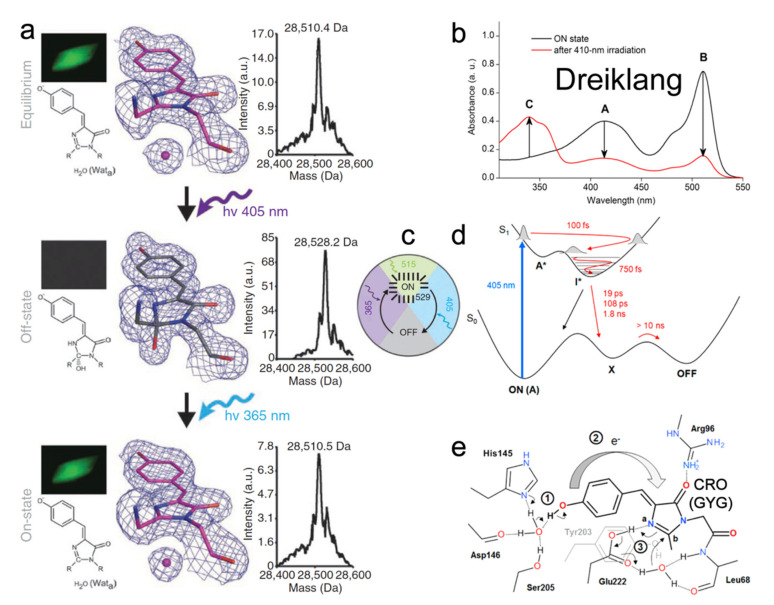
Photophysical characterization of the Dreiklang photoswitching. (**a**) Representative protein crystals with proposed chemical structures of the associated chromophores. Middle panels from top to bottom: X-ray structures with PDB IDs of 3ST2, 3ST3, 3ST4 [23]. For the equilibrium and on states, an I-ring-adjacent water molecule (Wat_a_, purple sphere) is separately shown. Right panels: representative ESI-MS spectra of Dreiklang photoswitched in solution, the mass change due to one water addition in the off state is conspicuous. (**b**) Steady-state absorption spectra of the on-(black) and off-state (red) of Dreiklang in aqueous buffer solution at pH 7.5. (**c**) Switching modality scheme for Dreiklang. (**d**) Schematic excited-state (S_1_) and ground-state (S_0_) potential energy surfaces during the on-to-off photoswitching with intermediates and associated time constants. X likely represents an anionic chromophore-water adduct state. (**e**) On-state chromophore H-bonding network with key surrounding residues, with arrows 1–3 depicting the proposed hydration event after electronic excitation. Adapted with permission from Ref. [23]. Copyright 2011 Nature America, Inc. Adapted with permission from Ref. [92]. Copyright 2017 American Chemical Society.

Similar to rsEGFP2, the ancestor of Dreiklang is also the jellyfish-derived wtGFP [66,90]. Four mutations (S65G, V68L, S72A, and T203Y) change the emission color of GFP from 513 to 527 nm and yield the yellow fluorescent protein (YFP) with a GYG chromophore (p*K*_a_ ≈ 7.0) [25]. The later Q69M mutation based on YFP led to Citrine that has a lower p*K*_a_ (5.7) and higher photostability [91]. Citrine fluoresces at ~529 nm with 516 nm excitation (FQY = 0.76), and it also displays the capability to switch with 400 nm to off state and 365 nm light to on state but only to a very small extent. To improve the switching contrast, the monomeric Dreiklang was developed with four mutations (V61L, F64I, Y145H, and N146D) based on Citrine [23]. In the native state, Dreiklang is in the on state with p*K*_a_ of 7.2 and two absorption peaks at 412 nm (neutral form) and 511 nm (anionic form) as shown in Figure 10b. Excitation at 511 nm yields fluorescence at 529 nm with an FQY of 0.41 and causes no photoconversion (Figure 10c). The 405 nm irradiation “bleaches” the protein, signified by the disappearance of both 412 and 511 nm peaks and rise of a new peak at ~340 nm (Figure 10b). Light irradiation at ~365 nm can switch the protein back on (Figure 10c). This switching pattern dramatically differs from both the negative and positive RSFPs (see Section 2 and Section 3 above) wherein the photoswitching light and fluorescence excitation light are always entangled; thus, making Dreiklang with an operational pH range of 6–9 across physiological temperatures (10–40 °C) highly beneficial for nanoscopy to avoid the tug-of-war between the on, off, and excitation light and also reduce the overall laser power needed to operate the RSFPs and perform high-resolution imaging [23].

To reveal the underlying mechanism, crystal structures of Dreiklang in the native, off, and on states were solved (Figure 10a) [23]. Unlike the common chromophore *cis*-*trans* isomerization in negative and positive RSFPs, the Dreiklang chromophore has *cis*-geometry regardless of its on/off states. Notably, the five-membered imidazolinone (I)-ring of the chromophore is hydrated (covalent addition of water) in the off state, which breaks/shortens the original π-conjugation across the P- and I-rings while leading to a distorted I-ring (Figure 10a, second row), resulting in a new blue absorption band at ~340 nm and concomitant decrease in two absorption bands at ~410 and 510 nm (Figure 10b, red trace). This observation was confirmed by the electrospray ionization mass spectrometry (ESI-MS) results: the mass of dark protein is ~18 Da (i.e., molecular weight of one water molecule) more than that in bright state (Figure 10a, right panels). Examining the H-bonding network near the chromophore revealed a conserved water molecule near the I-ring, also being stabilized by Y203 and E222 (Figure 10a, left and middle panels (Wat_a_); and Figure 10e). This water molecule likely participates in the chromophore hydration and dehydration events during reversible photoswitching. However, there are still unresolved questions. For instance, although a *trans*- or twisted chromophore does not exist in steady-state structures, it could appear or transiently exist in the excited state. Additionally, since both the neutral and anionic chromophores are in the *cis*-geometry, it is unclear why only the on-state neutral chromophore (~400 nm absorption) can undergo hydration and switch to an off state (see below).

In 2017, Lacombat et al. utilized fs-TA and studied the photobleaching process of Dreiklang [92]. From the fs-TA spectra upon 405 nm excitation of the neutral chromophore in pH 6.3 aqueous buffer, the initial decay of ~470–480 nm SE band (excited protonated species) and rise of a redder SE band around 520 nm (the intermediate deprotonated species) strongly indicate an ESPT process with ~100 fs time constant (Figure 10d) retrieved by global analysis. From sub-ps coherent spectral oscillations in TA signal of the longer-lived photoproduct anionic species (than the initial neutral species in the Franck–Condon region), a ~30–90 cm^−1^ mode was uncovered that is sensitive to the H/D isotope effect, attributable to an intermolecular modulation of the local H-bonding network that involves proton motions [89,93]. The authors surmised that ESPT of the *cis*-neutral chromophore leads to a charge transfer from the P-ring to I-ring and initiates a hydration process, while the adjacent E222 is protonated and can donate its proton to the C=N bond nitrogen on the I-ring and then facilitate the addition of hydroxide from water (Figure 10e). ESPT was thus proposed as the precursor for hydration, after which the anionic chromophore in the ground state (“X” generated on the ps-ns timescales, Figure 10d) needs to be re-protonated with protein environmental relaxation to reach the final dark state (“OFF” state, Figure 10d). However, this hypothesis was later challenged by computational studies [94,95]. Grigorenko et al. suggested that E222 is deprotonated in both on and off states, so it cannot donate a proton to the I-ring during on-to-off switch but instead acts as an effective proton shuttle with protonation state changes along the reaction profile [94]. Sen et al. proposed that ESPT occurs (but is strongly suppressed) in parallel with photobleaching, and the chromophore remains protonated throughout the switching process [95]. Their simulation results demonstrated that the light-driven chromophore in a locally excited (LE) state tends to relax into a charge transfer (CT) state, facilitated by electron transfer from Y203 to the chromophore. A neutral chromophore–Y203 radical pair can then form following the CT state wherein Y203 donates its hydrogen to the I-ring of the chromophore. After a back electron transfer from the chromophore to Y203, the positively charged I-ring (with C=N bond) is favored to undergo nucleophilic addition of hydroxide from water, concurrent with the reprotonation of Y203. The detailed photoswitching mechanism in the excited state is still under debate by active researchers in the field with different focuses and expertise. Vibrational spectroscopies (particular Raman spectroscopy that highlights the chromophore modes [57,62,96] with sensitivity to various protonation states of a particular chemical bond or functional group) are highly suitable to unravel this question. A caveat is that the exact PQYs of Dreiklang [23] or SPOON [24] are still unknown that may hinder future design and implementation of ultrafast techniques with subsequent spectral data analysis, which can be mitigated by high-level electronic structure and ab initio MD simulations.

## 5. Conclusions and Perspectives

It has been two decades since the discovery of the first RSFP [21]. To date, RSFPs have been extensively implemented in super-resolution imaging and promoted the development of many imaging techniques with exciting applications [97]. However, the fundamental understanding of their working mechanisms, especially the molecular processes occurring on ultrafast timescales (fs to ms), can still be considered an early stage. A series of ultrafast techniques, including the electronic and vibrational methods with UV, visible, IR laser pulses as well as X-ray crystallography, have been successfully implemented in studying RSFPs and significant insights have been gained, but there are still many outstanding questions.

There has been a consensus among the community that whether a photoswitching process can be investigated by ultrafast spectroscopy is highly dependent on the PQY. In this regard, the most studied process of RSFPs is the off-to-on photoswitching of negative RSFPs (e.g., Dronpa, rsEGFP2, and IrisFP) due to their associated large PQYs. The small on-to-off PQY of negative RSFPs, small bidirectional PQYs of positive RSFPs, and unknown PQYs of decoupled RSFPs currently hinder any proposed studies by an ultrafast experimental platform, especially concerning the electronic techniques that solely depend on the molecular species/state population change (also with intrinsically broad and overlapping transient electronic bands) [39,98]. In addition, most investigations have focused on the photoinduced on and off states, but the resting/native state is rarely studied, e.g., the low-pH-induced neutral form in a negative RSFP [78] and the native dark state of a positive RSFP. Since the native state determines the true starting point of a photoswitching event, resolving its structural dynamics properties will provide additional crucial insights into the natural evolution and rational bioengineering for more versatile RSFPs. Furthermore, for the targeted photoswitching improvements with fluorescence being intrinsically a ns event, one of the major research areas should focus on the ultrafast interplay between ESPT, chromophore conformational change, ring planarity change, and protein pocket flexibility. Based on the collective insights from previous literature reviewed herein, a fine balance needs to be achieved between the photoswitching speed and FQY for practical bioimaging applications because the chromophore cannot be too restricted (slow or no photoswitching) or too free (small FQY or nonfluorescent). In other words, just the right amount of freedom and restraints works best for an “ideal” RSFP, which can be rationally designed and effectively tuned for specific advances such as fatigue resistance, increased switching cycles, higher spatial resolution, redder excitation and emission wavelengths, lower switching light intensity, enhanced fluorescence switching contrast, fast switching speed, and high on-state brightness, just to name a few.

Regarding the ultrafast techniques, the most commonly used one is fs-TA which offers important insights into the photoinduced reaction timescales but lacks structural sensitivity. Vibrational spectroscopy like TRIR can detect the fingerprint of characteristic structural change of not only the chromophore but also the surrounding protein residues, which may pose considerable challenges in accurately assigning the highly overlapped vibrational peaks. The complementary technique, FSRS [57,62,65,89,99,100], which has not been specifically used to study RSFPs (except for one recent work on LEA that is IrisFP-like with our new findings [78]) but has been successful in investigating photoisomerization in retinal [101] and phytochromes [102], could be implemented especially considering that the large Raman/electric polarizability of a twisted chromophore may overcome the limitation of low transient populations during the RSFP photoswitching events. Furthermore, the dynamic resonance enhancement for transient Raman modes provides the extra experimental strategy that can effectively increase the signal-to-noise ratio of fleeting molecular species [39,89]. Recent examples for rational protein design on the basis of time-resolved spectroscopies, particularly on ultrafast timescales and also with femtosecond Raman signatures, have enabled the FP-based calcium sensors to acquire different or improved functions [62,103]. Meanwhile, fs X-ray crystallography can directly solve protein structures during photoswitching but can only focus on a limited number of time points, while the crystal lattice may cause notable differences from the physiologically relevant (native) environments such as aqueous buffer solution, as recently demonstrated for an altered photocycle of the photoactive yellow protein in crystalline and solution states [104]. Therefore, a strategic combination of these ultrafast techniques from table-top setups to large user facilities, in conjunction with quantum mechanics/molecular dynamics calculations in both the electronic ground and excited states (ideally throughout the entire photocycle) are highly desirable in future studies. Through such a powerful and fulfilling spectroscopic lens, the mechanistic insights to be gained, enriched, and interpreted will guide the rational design and efficient engineering of more RSFPs with higher PQYs, which in turn can benefit more ultrafast studies, thereby forming a positive feedback loop and efficient discovery cycle for next-generation RSFPs and life imaging breakthroughs.

## Data Availability

Not applicable.
